# Special Issue: Molecular Research in Epilepsy and Epileptogenesis

**DOI:** 10.3390/ijms26188795

**Published:** 2025-09-10

**Authors:** Stanisław J. Czuczwar

**Affiliations:** Department of Pathophysiology, Medical University of Lublin, 20-090 Lublin, Poland; stanislaw.czuczwar@umlub.pl

Epilepsy is a chronic neurological disease associated with brain pathology. Its most characteristic symptom is epileptic seizures [1]. The incidence of the disease is 5 to 10 cases per 1000 people in the general population, meaning that approximately 70 million people are affected worldwide [2,3]. There are two types of epileptic seizures in terms of onset, namely focal and generalized. The former involves one area of the brain at first and manifests in a particular region of the body, while the latter begins bilaterally in the brain, and involves cortical and subcortical brain areas [4]. Focal seizures may be simple (without loss of consciousness) or involve impaired awareness. In generalized tonic–clonic convulsions, the entire body is affected by seizure activity accompanied by loss of consciousness [4,5]. Non-convulsive seizures may also be distinguished, for instance absence seizures [2]. In approximately 40% of patients with epilepsy, a genetic etiology can be identified, while in the remaining patients the etiology may be symptomatic, e.g., as a result of an initial insult (brain injury, brain tumor, metabolic disorders or brain infection) [6]. Currently, the treatment for epilepsy includes pharmacological or neurosurgical methods and neurostimulation techniques [7]. About 50–60% of patients respond positively to the first antiseizure medication (ASM), which can fully inhibit seizure activity [7]. An alternative monotherapy or combination therapy with ASMs may increase the responder rate up to as much as 80% [7], and patients with pharmacoresistant epilepsy may be considered for neurosurgery. Neurosurgery is effective in 50–80% of individuals provided that the patient is properly selected based on, for example, the type of epilepsy, the location of the epileptogenic brain area or brain pathology [8]. For patients who are not candidates for neurosurgery or who have failed neurosurgery, neurostimulation techniques (e.g., vagus nerve stimulation or deep brain stimulation) can be used. These techniques are usually effective in reducing seizure burden by as much as 75% [9].

Some ASMs may act through the enhancement of GABA-mediated inhibition or by inhibiting glutamate-induced excitation. Others may target Ca^2+^, Na^+^, or K^+^ ion channels or entirely new targets such as inflammatory pathways or m-TOR [10]. However, the available ASMs have not been documented to effectively affect the process of epileptogenesis [11], which is responsible for transforming a healthy brain into an epileptic brain [11]. Only some ASMs (eslicarbazepine, lamotrigine or levetiracetam) may bear antiepileptogenic potential [11]. The process of epileptogenesis begins with the initial insult and consists of two phases; an acute phase, characterized by neurodegeneration, neuroinflammation and transcriptional events, lasting from hours to weeks, and a second chronic phase, lasting at least months, including, among other things, aberrant neurogenesis, mossy fiber sprouting and aberrant organization of neuronal circuits [11]. ASMs, by preferentially acting on ion channels and/or neurotransmitter-mediated events, may exert anticonvulsive activity in established epilepsy, ultimately leading to a reduction in seizure frequency or a complete cessation of seizure activity [7].

Approximately 30% of patients with epilepsy demonstrate drug resistance despite the use of combination therapies with multiple ASMs [7]. Rational polytherapy, based on synergistic ASM combinations, could probably reduce the percentage of drug-resistant cases further [7]. Nevertheless, the problem of drug-resistant epilepsy remains unsolved, even though more than 30 ASMs are available, some of which, with novel mechanisms of action, have been developed quite recently [10]. The currently used pharmacotherapy aims to alleviate seizure activity and, as mentioned above, its effectiveness in inhibiting epileptogenesis seems to be rather low [11].

The articles in this Special Issue address various aspects of epilepsy and epileptogenesis. These include data on neuroinflammation biomarkers for epileptogenesis, the genetic basis of epilepsy, oxidative stress in epilepsy, and novel compounds with potential as ASMs. The paper by Kovalenko et al., “Identification of reliable reference genes for use in gene expression studies in rat febrile seizure model”, addresses the most relevant reference genes in multiple brain regions of rats after neonatal febrile seizures. The results indicate that the *Ppia* gene showed the greatest expression stability and can be used for normalization in this seizure model. Abram et al. (“Development of novel alaninamide derivatives with anticonvulsant activity and favorable safety profiles in animal models”) analyzed a number of ((benzyloxy)benzyl)propanamide compounds in search of potential ASMs. One of them (compound **5**) exhibited potent anticonvulsant activity in maximal electroshock and 6 Hz seizure models with low adverse potential. Bifidobacterium breve strain A1 was tested against pentylenetetrazole-induced kindling in mice by Ishii et al. in “Oral administration of probiotic Bifidobacterium breve ameliorates tonic–clonic seizure in a pentylenetetrazole-induced kindling mouse model via integrin-linked kinase signaling”. The authors proved that the strain inhibited the seizure activity, which was associated with integrin-linked kinase-induced phosphorylation of Akt Ser^473^ in the hippocampus. Fukuyama et al. (“Age-dependent activation of pannexin 1 contributes to the development of epileptogenesis in autosomal dominant sleep-related hypermotor epilepsy model in rats”) provide evidence that the physiological ripple burst may become epileptogenic/ictogenic in patients vulnerable to autosomal dominat sleep-related hypermotor epilepsy. In a series of review articles, “The oxidative stress in epilepsy—Focus on melatonin”, Kamieniak et al. provide data on anticonvulsant effects of exogenous antioxidants and melatonin in animal seizure models. Melatonin and some exogenous antioxidants may be considered adjuvants in the management of epilepsy. Another review by Aguilar-Castillo et al., “A systematic review of the predictive and diagnostic uses of neuroinflammation biomarkers for epileptogenesis”, postulates that a number of cytokines and chemokines may play a significant role in the early diagnosis of drug-resistant epilepsy. An article by Borowicz-Reutt et al. (“Genetic background of epilepsy and antiepileptic treatment”) indicates the complexity of the protective activity of ASMs depending on gene mutations. For instance, gain-of-function mutations in genes encoding sodium channels determine the anticonvulsant effects of sodium channel blockers. However, loss-of-function mutations, on the other hand, have the opposite effect. The last article is a case report by Han et al., entitled “The aggravation of neuropsychiatric symptoms in the offspring of a Korean family with intellectual disability and developmental delay caused by a novel *ARX* p.Lys385Ter variant”, concerning the *ARX* gene, mutations of which lead to a range of neurological disorders, including epilepsy. The authors are of the opinion that this novel heterozygous *ARX* variant is most likely responsible for the patient’s intellectual disability, developmental delay, agenesis of the corpus callosum, and developmental epileptic encephalopathy.

It is very likely that with the available ASMs, we have already reached a point where the entire population of epilepsy patients will never be seizure-free. Indeed, ASMs may be considered purely symptomatic drugs that generally have nothing to do with the epileptogenesis process. Even if they could inhibit epileptogenesis, patients would be treated after epileptogenesis had already occurred. Thus, the strategy for the effective pharmacological treatment of epilepsy is expected to focus on inhibiting epileptogenesis. During epileptogenesis, significant changes occur in the expression of genes controlling a number of signaling pathways. Examples include genes for insulin growth factor-1, mammalian target of rapamycin (m-TOR), p-38 mitogen activated kinases or Janus kinase signal transducer and activator of transcriptional proteins [11]. To date, it has not been possible to create an antiepileptogenic treatment strategy in humans, although promising data emerge from preclinical studies. For instance, brivaracetam has been shown to prevent seizure activity in rats following traumatic brain injury [12]. A real breakthrough may occur in a relatively short time, as a number of antiepileptogenic trials have been already initiated on the preventive effects of esclicarbazepine acetate, perampanel or biperiden in patients with stroke or traumatic brain injury [12]. After all, there are a number of options for antiepileptogenesis based on animal models—for instance, the blockade of angiotensin II type 1 receptors, nonsteroidal anti-inflammatory drugs, antioxidants or antisense nucleotides [11,12]. Because oxidative stress, at least indirectly, may stimulate epileptogenesis, exogenous antioxidants (for example, resveratrol or sulforaphane) may possess antiepileptogenic potential. The data on melatonin are ambiguous [13]. Existing clinical trials, as mentioned above, assume that stroke or head trauma is very likely to initiate epileptogenesis, although only a proportion of patients with these conditions will develop epilepsy. A good example is that stroke patients who develop epilepsy are the range of 2–20% of cases [14]. The 10-year risk of developing posttraumatic epilepsy following focal cerebral injuries is approximately 13% and may be approximately 50% following penetrating head injury [15]. Therefore, in order to develop future antiepileptogenic treatments, an important issue regarding markers of epileptogenesis needs to be resolved. So far, a number of potential biomarkers indicative of epileptogenesis have been suggested based on electroencephalograhic, imaging, molecular and behavioral data, but their clinical use has not been confirmed [16]. The clinical validation of such biomarkers therefore seems to be a milestone for further studies aimed at developing antiepileptogenic therapies.
